# Anti-Nogo-A antibodies prevent vascular leakage and act as pro-angiogenic factors following stroke

**DOI:** 10.1038/s41598-019-56634-1

**Published:** 2019-12-27

**Authors:** Ruslan Rust, Rebecca Z. Weber, Lisa Grönnert, Geertje Mulders, Michael A. Maurer, Anna-Sophie Hofer, Andrea M. Sartori, Martin E. Schwab

**Affiliations:** 1 Institute for Regenerative Medicine, University of Zurich, 8952 Schlieren, Zurich Switzerland; 2 Dept. of Health Sciences and Technology, ETH Zurich, 8092 Zurich, Switzerland; 3 Dept. of Biology, ETH Zurich, 8093 Zurich, Switzerland

**Keywords:** Blood-brain barrier, Stroke

## Abstract

Angiogenesis is a key restorative process following stroke but has also been linked to increased vascular permeability and blood brain barrier (BBB) disruption. Previous pre-clinical approaches primarily focused on the administration of vascular endothelial growth factor (VEGF) to promote vascular repair after stroke. Although shown to improve angiogenesis and functional recovery from stroke, VEGF increased the risk of blood brain barrier disruption and bleedings to such an extent that its clinical use is contraindicated. As an alternative strategy, antibodies against the neurite growth inhibitory factor Nogo-A have recently been shown to enhance vascular regeneration in the ischemic central nervous system (CNS); however, their effect on vascular permeability is unknown. Here, we demonstrate that antibody-mediated Nogo-A neutralization following stroke has strong pro-angiogenic effects but does not increase vascular permeability as opposed to VEGF. Moreover, VEGF-induced vascular permeability was partially prevented when VEGF was co-administered with anti-Nogo-A antibodies. This study may provide a novel therapeutic strategy for vascular repair and maturation in the ischemic brain.

## Introduction

Ischemic stroke results from focal cerebral ischemia due to occlusion of a cerebral blood vessel. At the core of the stroke, affected brain regions lose their supply of oxygen and glucose with immediate disturbance of function followed by necrotic cell death within hours. Surrounding the core is a zone of reduced blood flow with partial blockade of vessels, the ischemic border zone. Prolonged ischemia leads to tissue damage in this zone; however, restoration of blood supply by angiogenesis, the formation of new blood vessels, may prevent some of these degenerative events^1,2^. Preclinical research has linked therapeutic enhancement of angiogenesis through vascular growth factors to improved neurological outcomes, but these therapies have failed to be translated into clinical application due to safety concerns^3,4^. In parallel to inducing angiogenesis, vascular growth factors have been shown to trigger a cascade of aggravating events including increased vascular permeability and blood brain barrier breakdown as well as an increased risk of bleedings from immature, unstable vessels (hemorrhagic transformation)^5–8^. VEGF has been shown to disrupt the barrier function of the vessel endothelium through the promotion of pericyte detachment and degradation of tight junction proteins including claudins^8^ and cadherins^9^. Moreover, VEGF delivery appears to have a narrow therapeutic window due to dosing and a half life time of only several minutes *in vivo*^10,11^. Thus, alternative strategies are required without the risk of vascular leakage and bleeding.

Recently, the neurite outgrowth inhibitor Nogo-A, a membrane protein expressed by oligodendrocytes and subpopulations of neurons, has been shown to limit vascular growth in development^12^ and after CNS ischemia^13^. Its neutralization improved vascular repair in the ischemic border zone of cerebral strokes as well as functional outcome.

Here, we hypothesized that Nogo-A might represent a safe pro-angiogenic therapeutic target that does not increase vascular permeability and BBB integrity in contrast to VEGF. We identified anti-Nogo-A antibodies as promising new reagents to enhance vascular repair and maturation. In particular, we demonstrate that anti-Nogo-A antibodies (1) have similar pro-angiogenic effects as local VEGF treatment (2) do not increase vascular permeability in the peri-infarction regions, (3) partially reverse the VEGF induced blood vessel leakage when co-administrated.

## Results

### Blood-brain-barrier permeability is elevated in peri-infarct regions following ischemic stroke

**Figure 1:**
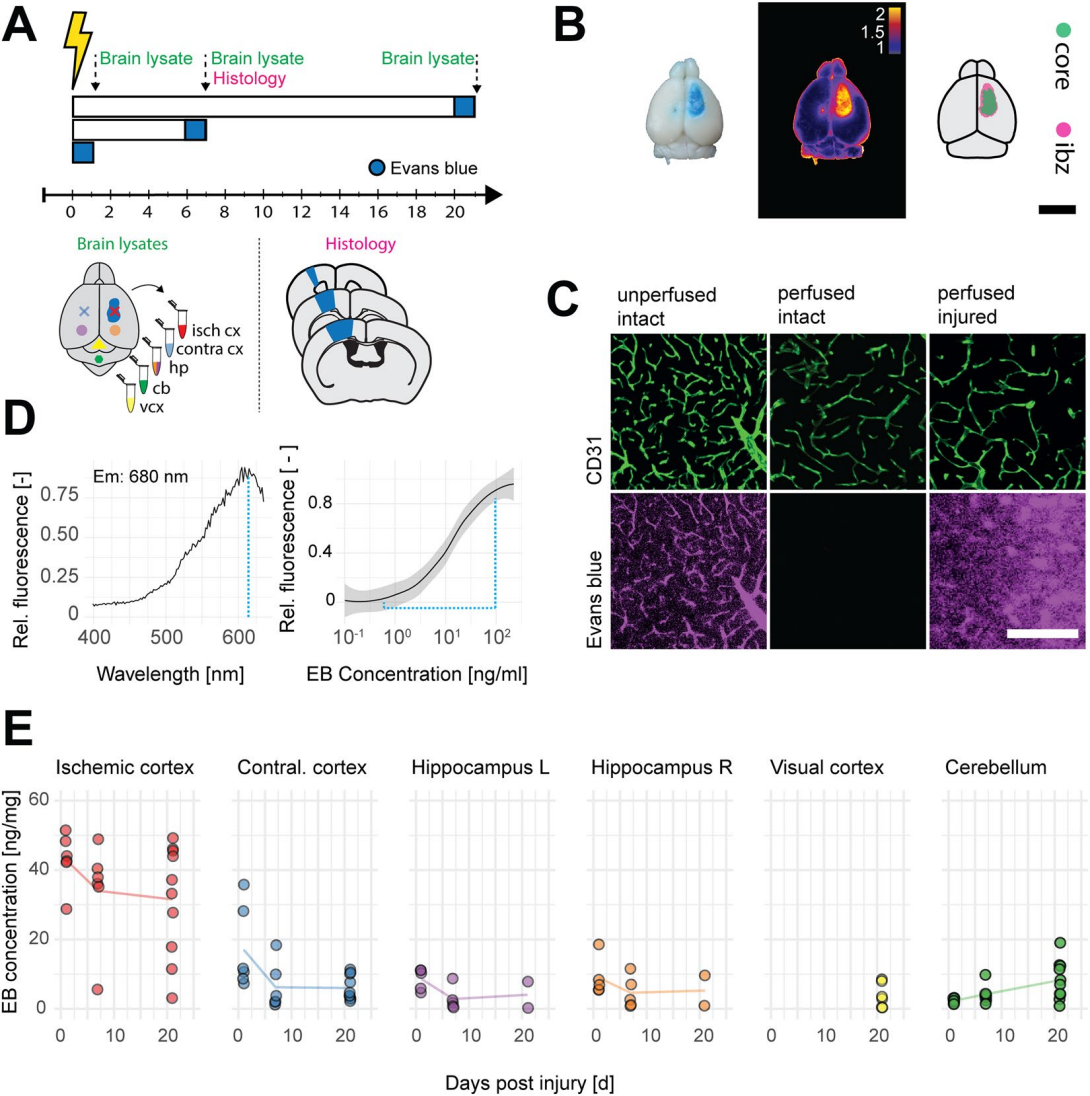
Temporal evolution of blood brain barrier disruption in brain lysates. (**A**) Schematic representation of the experimental timeline and the dissected brain regions for EB measurement. (**B**) Representative stroked brain with incorporated EB dye as RGB, heatmap and binarized image, scale bar: 5 mm. (**C**) Microscope images showing EB dye accumulation in non-perfused vasculature (CD31), its absence in intact perfused vessels, and its leakage into the injured peri-infarct parenchyma. Scale bar 100 um. (**D**) Absorbance spectrum and spectroscopic detection limit shown by EB dilution curve. (**E**) Quantitative evaluation of EB signal intensities in brain lysates of different regions from day 1 to day 21 after stroke including the injured and contralesional cortex, hippocampus, cerebellum and visual cortex. Isch cx; ischemic cortex, contra cx; contralesional cortex, hp; hippocampus, cb; cerebellum, vcx, visual cortex. EB; Evans Blue, ibz; ischemic border zone.

A significant hallmark of the brain tissue in the peri-infarct regions is the dysfunction of the BBB resulting in edema and the penetration of serum proteins into the CNS parenchyma. Yet the role of disturbed vascular permeability in stroke progression is not clear. We induced a photothrombotic stroke in the sensorimotor cortex of adult mice and analyzed vascular permeability in the acute (1 day post injury (dpi)), subacute (7 dpi), and chronic (21 dpi) phase by Evans Blue (EB) extravasation in micro-dissected lysates of different brain regions (Fig. 1A,B). Moreover, histology of coronal cortical sections was performed in a subgroup of animals that were either intact or at 7 dpi in the peri-infarct regions (Suppl. Figure S1). In the intact animals, EB was entirely confined to the vessel system in the CNS and could be fully removed by perfusion (Fig. 1C). EB/albumin concentrations in lysed brain tissue could be detected (at Ex: 620/Em: 680) with a sensitivity of 0.006 ng/mg (LOD), or respectively 0.019 ng (LOQ) per mg brain lysate (Fig. 1D). Analysis of EB extravasation in the peri-infarct region revealed a disrupted BBB throughout the time course (1 dpi: 42.91 ± 7.80 ng/mg, 7 dpi: 34.01 ± 14.77 ng/mg, 21 dpi: 31.54 ± 16.06 ng/mg tissue compared to uninjured cortices 5.32 ± 0.01 ng/mg, all p < 0.001, Fig. 1C,E). With progressing time the intragroup variability increased, suggesting a partial restoration of the BBB in individual animals. Interestingly, acutely after stroke, a subset of animals showed increased EB concentrations also in the contralesional cortex (17.04 ± 11.92 ng/mg tissue, p < 0.001) and in both hippocampi (left: 8.63 ± 3.09 ng/mg, p = 0.002, right: 8.97 ± 5.49 ng/mg, p = 0.027) compared to corresponding regions in uninjured animals, suggesting the extension of the BBB damaging effects into the contralesional hemisphere (Fig. 1E). Little to no signal (<10 ng/mg tissue) was detected in visual cortex and cerebellum through the time course compared to uninjured controls (p > 0.05) (Fig. 1E).

These results show a massive breakdown of the BBB from d.1 to d.21 after stroke, which is mostly restricted, however to the peri-infarct cortex in addition to the stroke core.

### Antibody-mediated neutralization of Nogo-A and vascular endothelial growth factor application display comparable angiogenic effects in the ischemic brain

BBB damage with subsequent edema and inflammation following stroke and other cerebrovascular diseases result in neuronal damage and prolonged loss of brain functions^14–17^. Therapeutics like VEGF, which enhance vessel growth but also lead to BBB opening therefore have only limited translational value^5,7,9^. We evaluated an alternative pro-angiogenic strategy by blocking the inhibitory factor Nogo-A with antibodies^18^ and studied the effects of this treatment on the BBB in the peri-infarct region surrounding the stroke.

**Figure 2:**
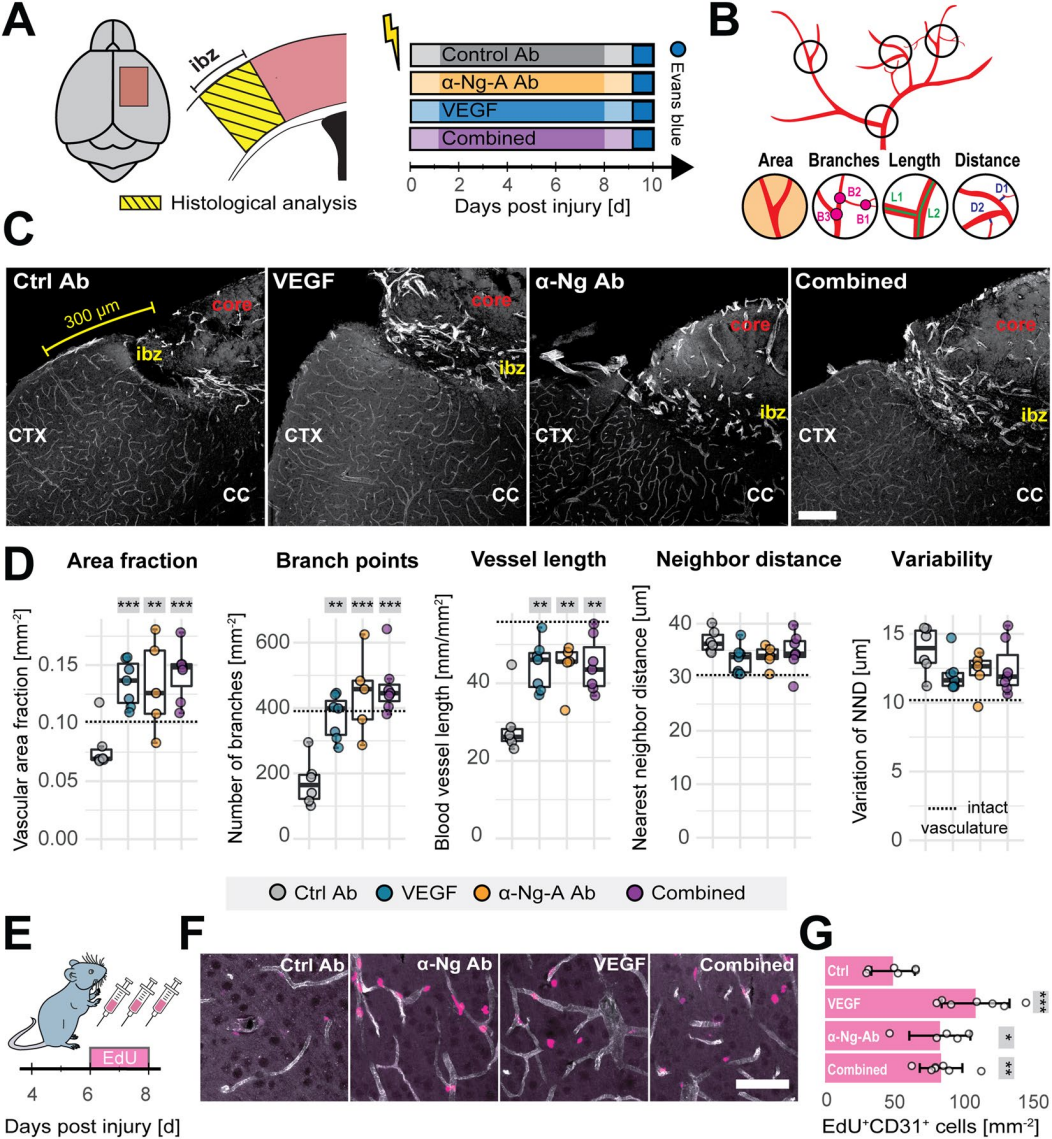
Vascular repair in the peri-infarct region following stroke in response to VEGF and anti-Nogo-A antibodies. (**A**) Schematic representation of the analyzed cortical region and experimental timeline in the four experimental groups: Ctrl-Ab (N = 6), VEGF (N = 7), α-Ng-Ab (N = 5), Combined (VEGF and α-Ng-Ab) (N = 7). (**B**) Overview of the assessed vascular parameters including the vascular area fraction (area), number of branch points (branches), vessel segment length (length) and nearest neighbor distance and variability (distance). (**C**) Fluorescent images of blood vessels (CD31^+^) in the peri-infarction region in the four treatment groups. Scale bar = 100 μm. (**D**) Quantitative evaluation of the vascular area fraction, number of branches, vessel segment length, nearest neighbor distance and variability in the peri-infarct region. (**E**) Experimental timeline of EdU injections. (**F**) Fluorescent images of newly formed blood vessels (EdU^+^ - pink; CD31^+^ - white), Scale bar = 50 μm. (**G**) Quantification of EdU^+^/CD31^+^ cells in the peri-infarct region. Data are represented as mean ± SD. Each dot in the plots represents one animal and significance of mean differences between the groups was assessed using Tukey’s HSD. Asterisks indicate significance: *P < 0.05, **P < 0.01, ***P < 0.001. CTX, cortex; CC corpus callosum.

Initially, we compared the pro-angiogenic effects of a 7 day intraventricular infusion of either VEGF, or anti-Nogo-A antibody (α-Ng-Ab), or a combined administration of VEGF and α-Ng-Ab (Comb), or a) control antibody (Ctrl-Ab) (Fig. 2A). In line with previous observations, Ctrl-Ab receiving animals showed a large drop in vessel density, branch number and vessel length within the peri-infarct region up to 300 µm from the border of the stroke core compared to the contralesional hemisphere^18^ (Fig. 2D). In contrast, the VEGF, the α-Ng-Ab, and the Comb groups displayed considerable improvement in vascular repair (Fig. 2B-D). The vascular area fraction in the region around the stroke core was increased more than 1.5-fold (VEGF: 0.134 ± 0.019, p = 0.004; a-Ng-Ab: 0.132 ± 0.039, p = 0.012; Comb: 0.143 ± 0.023, p = 0.001) compared to controls (Ctrl-Ab: 0.078 ± 0.019). The number of branches per mm^2^ increased by 114% (VEGF, 371.84 ± 66.67, p = 0.003), 156% (α-Ng-Ab, 443.69 ± 127.91, p < 0.001), and by 168% (Comb, 464.97 ± 85.39, p < 0.001) compared to control (Ctrl-Ab:173.20 ± 71.04) and the length of blood vessel segments per mm^2^ increased by 53% (VEGF, 44.44 ± 6.29 mm, p = 0.004), 53% (α-Ng-Ab, 44.22 ± 6.44 mm, p = 0.008) and by 54% (Comb: 44.70 ± 7.12 mm, p = 0.003) compared to control (Ctrl-Ab: 28.95 ± 7.89 mm). Nearest vessel neighbor distance (NND) and variability in the distribution of the blood vessels revealed no significant differences (Fig. 2D). The combined treatment did not show any beneficial angiogenic effects compared to either VEGF (area fraction: +6%, branching: +25%, length: +0.5%, NND: +4%, all p > 0.05) or a-Ng-A Ab (area fraction: +8%, branching: +5%, length: +1%, NND: +3%, all p > 0.05) alone.

Importantly, no differences in total stroke volumes have been observed between the groups (Suppl. Figure S2).

In order to identify newly formed blood vessels, the nucleotide analogue, 5-ethynyl–2′-deoxyuridine (EdU) was given daily on days 6–8 after ischemia (around the peak of post-stroke angiogenesis^19^). Nuclei of CD31^+^ vascular endothelial cells that incorporated EdU were identified as newly formed after injury and quantified (Fig. 2 E, F). The number of EdU^+^/CD31^+^ cells per mm^2^ was significantly increased in the peri-infarct region of animals in treated with VEGF:109.84 ± 24.73, p < 0.001, α-Ng-Ab: 83.78 ± 22.31, p = 0.045 or the Comb group: 84.65 ± 15.50, p = 0.023) compared to control antibody treated mice (49.58 ± 15.87, Fig. 2G).

These data demonstrate a pro-angiogenic effect of α-Ng-Ab comparable to the effect exerted by VEGF. The combined treatment yielded equivalent results in terms of vascular repair and formation of new vessels.

### Nogo-A neutralization does not increase vascular permeability and counteracts VEGF-induced leakage in the peri-infarct cortex

**Figure 3:**
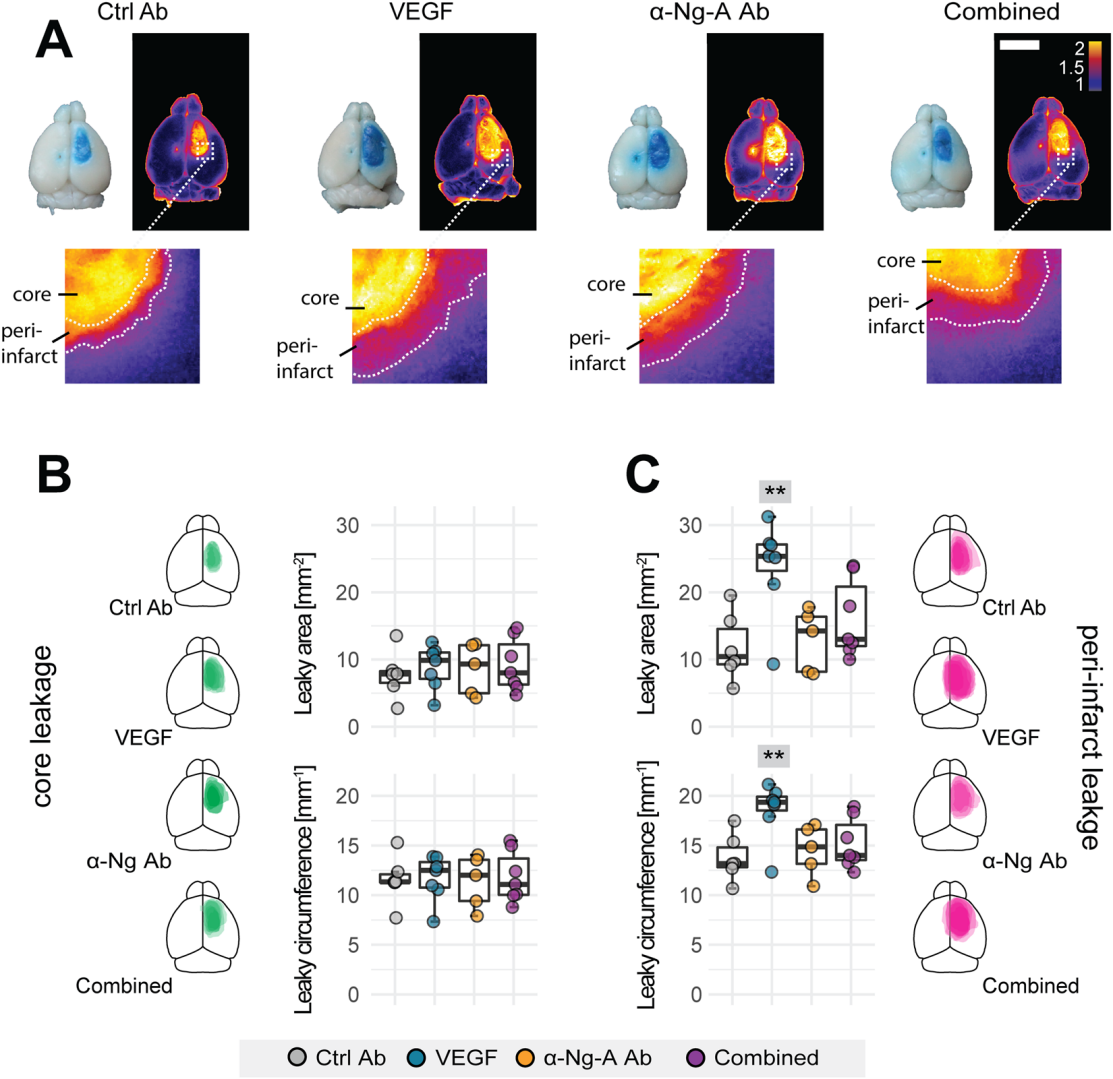
Vascular leakage in the peri-infarct region in response to VEGF or anti-Nogo-A antibodies in whole brain images. (**A**) Dorsal view of representative brains with extravasated EB in and around the stroke cores with corresponding heatmap. Scale bar: 5 mm (**B**) Quantitative measure of area (top) and circumference (bottom) of the EB leakage in the stroke core (**C**) and in the peri-infarct zone. Brain templates visualize local EB distribution from single animals in the core (green) and peri-infarct zone (purple). Data are represented as mean ± SD. Each dot in the plots represents one animal and significance of mean differences between the groups was assessed using Tukey’s HSD. Asterisks indicate significance: *P < 0.05, **P < 0.01, ***P < 0.001.

To examine the effects of VEGF and anti-Nogo-A treatment on BBB leakage, EB was systemically administered at 9 dpi; 24 hours prior to perfusion. Whole stroke brains were imaged and levels of tissue-bound EB were determined by signal intensity measurements. A strong EB signal was observed within the stroke core. In the peri-infarct zone the EB signal gradually decreased as distance from the stroke core in all experimental groups (Ctrl-Ab, VEGF, α-Ng-Ab, Comb; Fig. 3A). Quantitatively, levels of vascular leakage were assessed by measuring the circumference (circ.) and the area of signal intensities in the core zone (defined as >2x the intensity measured in the contralateral hemisphere) and in the peri-infarct regions (>1.5x intensity of the contralesional side. All groups showed comparable levels of core area in mm^2^ (Ctrl Ab: 7.74 ± 3.51, VEGF: 8.86 ± 3.24, α-Ng-Ab: 8.61 ± 3.81; Comb: 9.21 ± 3.95, all p > 0.88) and core circumference in mm (Ctrl Ab: 11.55 ± 2.43; VEGF: 11.70 ± 2.30; α-Ng-Ab: 11.38 ± 2.65; Comb: 11.83 ± 2.58, all p > 0.98) (Fig. 3B). However, animals treated with VEGF showed a markedly larger area of elevated extravascular EB levels within the peri-infarct region compared to the control cohort (Fig. 3C). Both the area (control: 11.82 ± 4.98; VEGF: 23.80 ± 7.05, p = 0.006) and the circumference (control: 13.76 ± 2.36; VEGF: 18.53 ± 2.91, p = 0.017) of elevated extravasated EB dye were significantly increased within the peri-infarct cortical region compared to the control cohort. In contrast, no increase of EB extravasation area in mm^2^ or circumference in mm was detected between the control (area: 11.82 ± 4.98, circ.: 13.76 ± 2.36), the anti-Nogo-A antibody group (area: 12.89 ± 4.61, p = 0.99; circ.: 14.52 ± 2.55, p = 0.96) and, interestingly, also the combined VEGF and α-Ng-Ab group (area: 16.09 ± 5.83, p = 0.56; circ.: 15.21 ± 2.57, p = 0.75, Fig. 3C).

**Figure 4:**
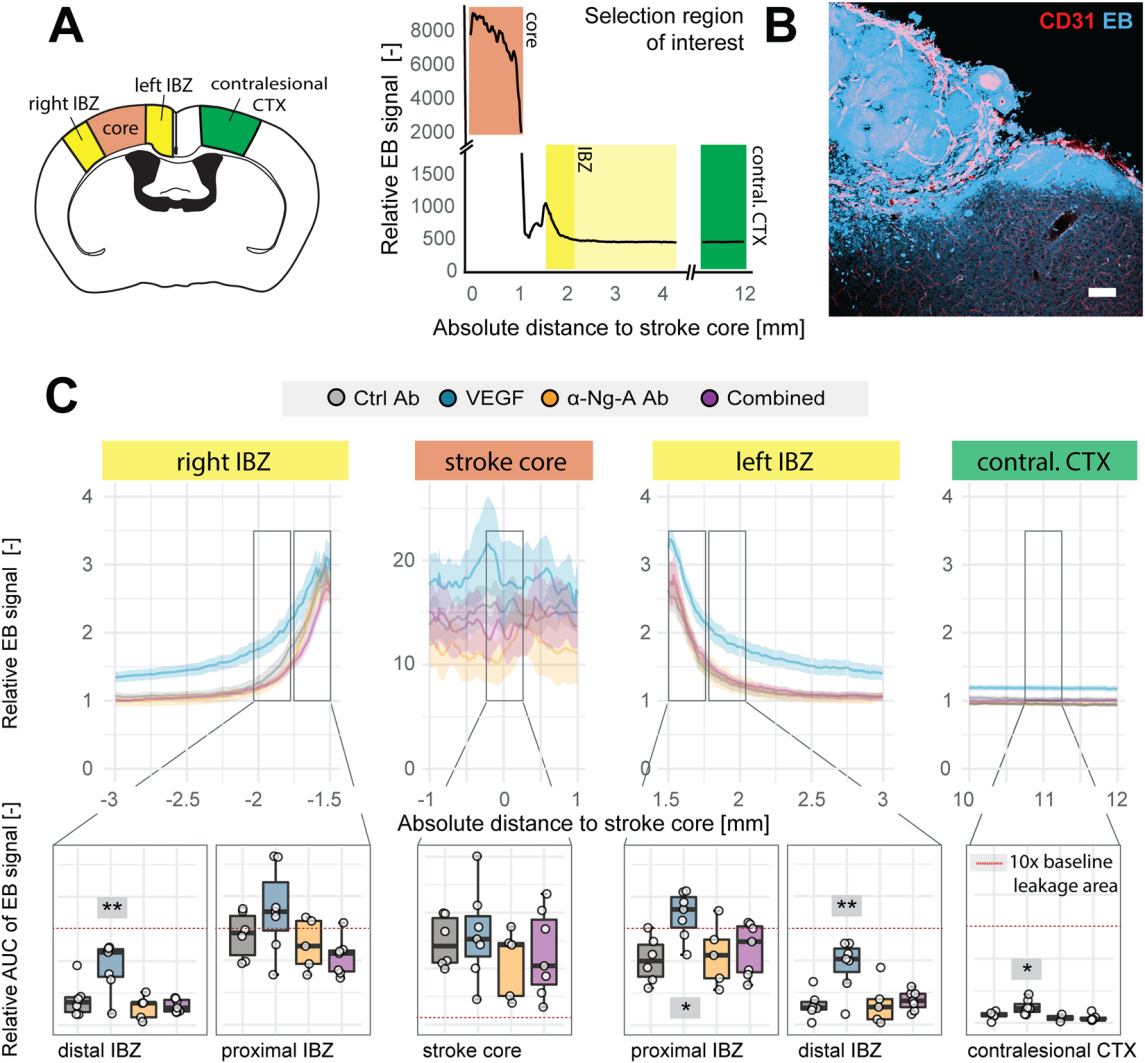
Spatial distribution of Evans Blue in cortical brain sections. (**A**) Schematic overview of regions of interest: 1) stroke core (red), proximal and distal peri-infarct regions (yellow), and contralesional site (green). Fluorescence intensities along the injured cortex are represented. Blood vessels are visualized by CD31 (red) immunostaining and leakage is visualized by EB fluorescence (blue). Scale bar = 50 μm (**B**) Relative EB signal intensities of the different treatment groups along the injured cortex (**C**) Quantitative evaluation of EB signal intensities as area under the curve. Data are represented as mean ± SD. Differences between the groups were assessed using Tukey’s HSD. Asterisks indicate significance: *P < 0.05, **P < 0.01, ***P < 0.001.

A histological analysis of vascular permeability was performed on brain sections for the stroke core, peri-infarct regions and the contralesional cortex (Fig. 4A). We observed a high EB signal in the stroke core (0–1 mm, Fig. 4A,B). Next to the core, we often observed a characteristic drop of the signal due to a frequent histological artefact: disruption of the tissue between the stroke core and the remaining parts of the brain. The second, lower peak of the EB signal was assigned to the peri-infarct ischemic border zone. Enhanced EB signal was detectable up to 0.6 mm from the stroke border (IBZ, 1.5–2.1 mm). Further away from the ischemic border zone the EB signal approaches that of the contralesional cortex (>2.1 mm) (Fig. 4B).

Differences between the treatment groups were assessed by calculating the area under the curve (AUC) normalized to the extent of signal at the contralesional cortex of Ctrl Ab animals. The signal intensities in the stroke core were comparable between all treatment groups (Fig. 4C). However, VEGF-treated animals displayed significantly increased signal intensities within both more proximal (±1.5–1.8 mm distance from stroke core) (right: +28.41 ± 51.96%, p = 0.533, left: +68.81 ± 36.19%, p = 0.031) and distal peri-infarct regions (±1.8–2.1 mm distance from stroke core) (right: +303.46 ± 30.34%, p = 0.001; left: +126.40 ± 33.25%, p = 0.014) compared to Ctrl Ab treated animals. Animals receiving either anti-Nogo-A antibodies or the combined treatment did not show any indication of increased BBB permeability within both proximal and distal peri-infarct regions compared to the control. Interestingly, VEGF treated animals also revealed enhanced vascular leakage on the contralesional hemisphere compared to all other groups (Ctrl-Ab: +179.59 ± 10.01%, p = 0.027; α-Ng-Ab: +134.59 ± 9.54%, p = 0.001; Comb: + 314.60 ± 9.39%, p = 0.005) (Fig. 4C).

These results show that the pro-angiogenic effect of VEGF is accompanied by enhanced vascular leakage in peri-infarct regions that are associated with vascular growth as well as in non-injured CNS regions. These leakage effects were not observed in anti-Nogo-A treated animals and could be partially prevented in a combined treatment.

### Nogo-A neutralization leads to the formation of a mature vascular network as opposed to VEGF treatment in the peri-infarct cortex

**Figure 5:**
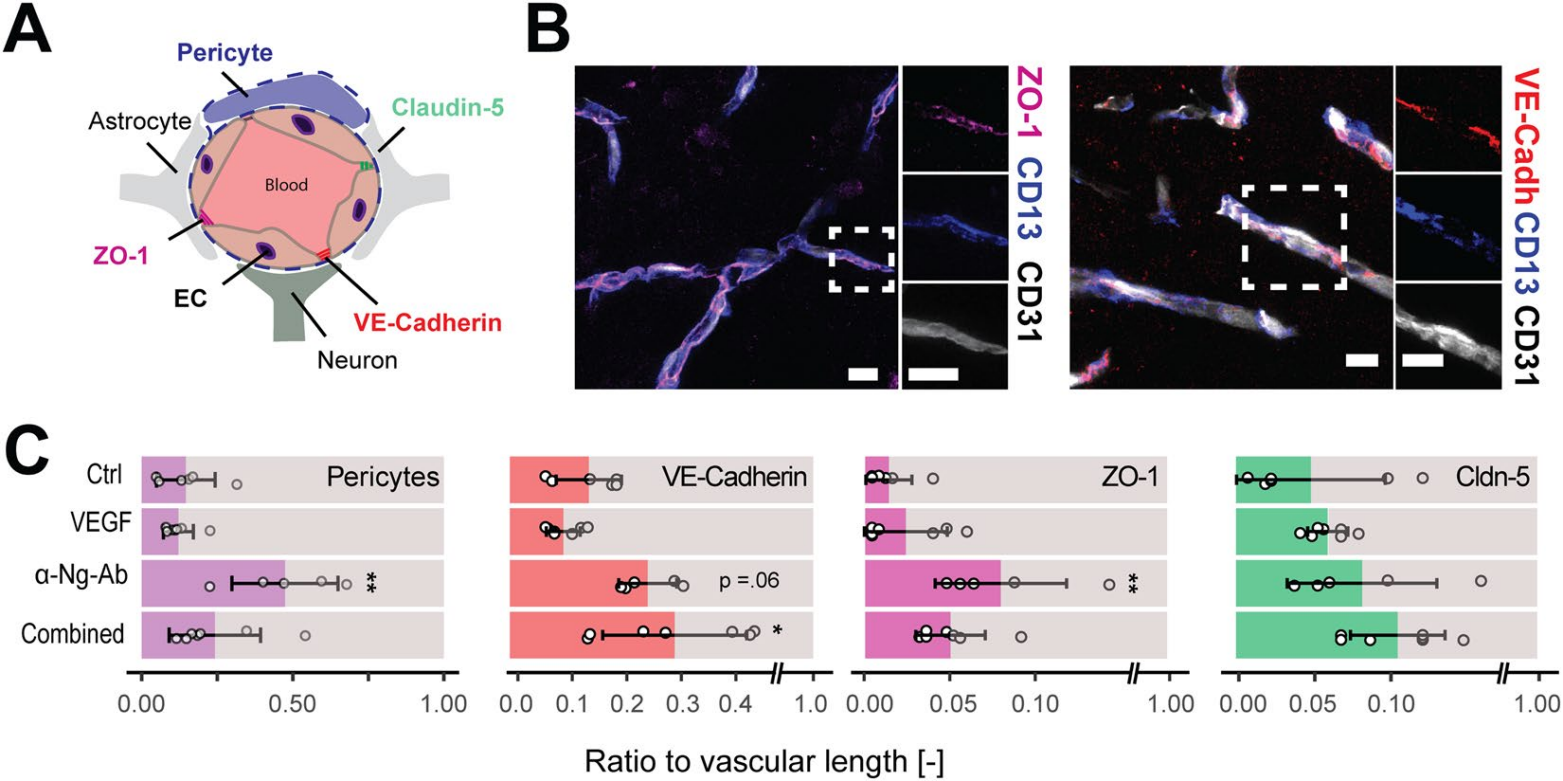
Maturation and tight junction composition of the endothelium in the ischemic border zone. (**A**) Schematic composition of blood brain barrier with the vasculature, pericytes, astrocyte end feet, neurons and the tight junction proteins (VE-Cadherin, ZO-1, Claudin-5). (**B**) Fluorescence images of the vascular endothelium with pericytes (CD13) and tight junction proteins (ZO-1 and VE-Cadherin). Scale bar = 5 µm. (**C**) Quantitative assessment of endothelial coverage with pericytes and tight junction proteins in the peri-infarct region. Data are represented as mean ± SD. Differences between the groups were assessed using Tukey’s HSD. Asterisks indicate significance: *P < 0.05, **P < 0.01, ***P < 0.001.

It has been previously observed that VEGF may induce a failure of recruiting periendothelial cells such as pericytes as well as a decrease of the function of tight junctions, e.g. by a drop in tight junction proteins (e.g. VE-Cadherin, ZO-1 and Claudin-5). We confirm a lower pericyte coverage of blood vessels in the IBZ of VEGF treated animals compared to those receiving anti-Nogo-A Ab (VEGF: 12.13 ± 4.97%; α-Ng-Ab: 47.50 ± 17.60%, p < 0.001, Fig. 5A-C). Moreover, the expression of tight junction proteins VE-Cadherin and ZO-1 were increased in anti-Nogo-A antibody treated animals (VE-Cadherin: VEGF: 9.25 ± 2.92%; α-Ng-Ab: 23.91 ± 5.03%, p = 0.007, and ZO-1: VEGF: 2.31 ± 2.35%; α-Ng-Ab: 7.71 ± 3.79, p = 0.01). The VEGF effects were partly reversed in the combined treatment (VE-Cadherin: Combined: 28.59 ± 12.49%, p = 0.004, ZO-1: 4.85 ± 1.99%, p = 0.15) (Fig. 5C). No significant differences were observed for Claudin-5 between the groups (Ctrl: 4.95 ± 4.94%, VEGF: 6.05 ± 1.33%, α-Ng-Ab: 8.33 ± 4.95% Combined: 10.68 ± 3.12%, p > 0.05) (Fig. 5C).

These results indicate that Nogo-A neutralization leads to the formation of a more mature vascular network and may prevent immature blood vessel formation caused by sole VEGF treatment. Conclusively, the study suggests that Nogo-A targeted therapy might represent a safe and effective alternative to VEGF treatment in terms of promoting vascular repair without the risk of blood brain barrier disruption following cerebral ischemia.

## Discussion

Angiogenesis and repair of the compromised vasculature in the stroke affected CNS tissue is a promising therapeutic target for post-stroke recovery. However, the clinical usability of vascular growth factors to promote revascularization of the ischemic tissue is compromised by the BBB destabilizing effects of VEGFs, which lead to enhanced edema and raise the risk of hemorrhagic transformation. In this study, we pursued an alternative strategy to enhance vascular repair by targeting the growth inhibitory protein Nogo-A. We demonstrate that antibody-mediated neutralization of Nogo-A protects the adult vasculature against leakage during the recovery phase after stroke while promoting vascular repair in the peri-infarct region. In particular, we show that 1) Nogo-A neutralization and VEGF treatment display equivalent pro-angiogenic effects by increasing vascular density in the peri-infarct regions; and 2) that newly formed vessels after Nogo-A neutralization are mature and do not show enhanced leakage as opposed to VEGF. Overall, this study identifies Nogo-A antibodies as a new and safe therapeutic agent to enhance angiogenesis following ischemic stroke.

Pro-angiogenic therapeutics have attracted increasing attention in the field of CNS ischemia. In particular, VEGF is well known for its prominent role in angiogenesis; however, VEGF therapy has failed to meet its promising expectations mainly due to safety concerns^5,20,21^. Our study supports these findings and shows that intracerebroventricular administration of recombinant VEGF for 7 days following a focal cortical stroke exacerbates vascular leakage, along with a prominent angiogenic effect. Besides its neuroprotective and pro-angiogenic effects^5,22^, VEGF has been previously shown to disrupt vascular barrier function and promote edema development^5,20,21^. Furthermore, newly formed VEGF-induced blood vessel sprouts are leaky and enhance inflammatory processes in the peri-infarct region. The decreased vessel stabilization has been linked to VEGFR2 activation leading to internalization of tight junction proteins (e.g. VE-cadherins) and loosening of endothelial cell contacts *in vitro* and *in vivo*^23^. Moreover, VEGF treatment may lead to pericyte ablation and inhibition of vascular smooth muscle function^24^. These detrimental effects have also been observed in other major ischemic conditions including myocardial infarction and retinal ischemia^25,26^. Consequently, VEGF treatment following ischemia raises considerable safety concerns and thus has not been implemented in human trials.

One key mechanism for VEGF-induced hyperpermeability is the RhoA/ROCK pathway; Rho kinase activity has strong barrier-disruptive effects and has been associated with increased VEGF levels following ischemia^27^. Interestingly, the vascular growth inhibitory signaling of Nogo-A via its receptor S1PR2 has been shown to also activate the RhoA/ROCK pathway^12,28^. Consequently, antibody-mediated inactivation of Nogo-A signaling may directly decrease RhoA levels in stroked tissue and contribute to the vascular integrity. Genetic and pharmacological blockage of S1PR2 reduced the neurovascular injury and permeability in experimental stroke^29^; previous work from our group confirmed these results^18^. The reduction of leakage following anti-Nogo-A antibody treatment may be a consequence of the higher number of pericytes and tight junctions in the vasculature of the peri-infarct region observed in our study. Interestingly, co-delivery of VEGF and anti-Nogo-A antibodies also reduced vascular hyperpermeability in the peri-infarct region and in part improved the maturation of the vasculature. However, it remains unclear if this is mediated through the Rock/RhoA pathway. Future studies may address the underlying mechanism between Nogo-A and VEGF following stroke and elucidate how anti-Nogo-A antibodies stabilize the microvasculature. Interestingly, the pro-angiogenic effects in the combined treatment group were not enhanced compared to the single treatment groups of VEGF or anti-Nogo-A antibodies alone. However, it is unclear whether this is due to intrinsic regulatory mechanisms that may inhibit excessive levels of angiogenesis or a consequence of the interaction between both substances.

A limitation of the study is the usage of end point measures and the photothrombotic stroke model. We cannot distinguish if the enhanced Evans blue signals in due to the damaged peri-infarct vasculature caused by the initial stroke injury or rather reflects the leakiness of newly formed and immature blood vessels. Dissecting these mechanisms is however, difficult and might be addressed in future studies with advancements in genetic models and *in vivo* microscopy.

Apart from Nogo-A, guidance molecule targeted therapies have been previously shown to act on both vascular repair and permeability following stroke. For example, Semaphorin 3A has been shown to suppress peripheral and CNS angiogenesis and to act as an important vascular permeability factor independent of VEGF^30,31^. Moreover, ephrinA1- EphA2 signaling has important roles in maintaining tight junction formation and its misregulation has been linked to BBB disruption^32^. Since it is known that genetic deletion of Nogo-A may change expression of other guidance molecules^33^, we cannot exclude that such indirect effects may also contribute to the reduced vascular leakage in anti-Nogo-A antibody treated animals.

Taken together, the administration of anti-Nogo-A antibodies may represent a new, promising, and safe therapeutic strategy to enhance angiogenesis, prevent vascular leakage and retain tissue integrity and functionality in the peri-infarct zone following ischemic stroke. Safety and feasibility of intrathecal anti-Nogo-A antibody delivery have recently been demonstrated in a phase I clinical trial for spinal cord injury^34^.

## Methods

### Experimental design

The goal of the present study was to test the angiogenic potential and safety of intracerebroventricularly applied anti-Nogo-A antibodies in mice with cerebral ischemia. We hypothesized that 1) pro-angiogenic effects caused by neutralization of Nogo-A are comparable to those exerted by VEGF and that 2) Nogo-A neutralization will not enhance vascular leakage when administered in the acute phase of an ischemic stroke unlike VEGF; thus making it a more suitable candidate for therapeutic angiogenesis following cerebral ischemia.

To evaluate the evolution of BBB opening following ischemic injury, we performed a time-course study. BBB opening was assessed at 1 day (N = 6), 7 days (N = 6) and 21 days (N = 10) after photothrombotic stroke; Evans blue i.v. was used as indicator of BBB permeability. Based on these findings and previous observations, we decided to assess vascular repair and permeability at 10 days following injury. Stroked mice received a continuous infusion of either VEGF (N = 7), anti-Nogo-A antibodies (N = 5), a combination of both (VEGF+ anti-Nogo-A Ab, N = 7) or a control antibody (N = 6) for seven consecutive days. The nucleotide analogue EdU was injected to detect newly formed blood vessels at day 6–8. One day before perfusion, animals were systemically injected with Evans blue. To characterize the loss of BBB integrity after stroke, we histologically and spectrophotometrically analyzed affected ischemic brain tissue. The mortality rate during the stroke surgeries was as expected at 5% in total. For the time course experiment mortality was 0% (0/22); during the BBB permeability experiment between Ctrl Ab, VEGF, a-Ng-A ab, combined, we observed a mortality of 3/28 animals: 2 animals died that received a-Ng-A ab, 1 animal died that received Ctrl-Ab. All animals are presented in the study; no statistical outliers were excluded. Data was acquired blinded.

### Animals

All animal experiments were performed in accordance with governmental, institutional (University of Zurich), and ARRIVE guidelines and had been approved by the Cantonal Veterinary Department of Zurich. Adult male wildtype mice (10–14 weeks) of the C57BL/6 strain (16–25 g) were used. Pilot experiments in adult female mice did not show any gender-specific differences (data not shown). Mice were housed in standard Type II/III cages at least in pairs in a temperature and humidity controlled room with a constant 12/12 h light/dark cycle (light on from 6:00 a.m. until 6:00 p.m.).

### Photothrombotic stroke and angiogenic treatment

Animals were deeply anesthetized with 5% isoflurane (Attane, Provet AG) in a transparent induction chamber. Stroke surgery was performed under 2–3% isoflurane. A photothrombotic stroke to unilaterally lesion the sensorimotor cortex was induced on the right hemisphere, as previously described^35^. Briefly, animals were fixed in a stereotactic frame (David Kopf Instruments) and the skull was exposed through a midline skin incision. A cold light source (Olympus KL 1500LCS, 150 W, 3000 K) was positioned over the right forebrain cortex at anterior/posterior: −1.5 mm to +1.5 mm and medial/lateral 0 mm to +2 mm relative to Bregma. Rose Bengal (10 mg/ml, in 0.9% NaCl, Sigma) was injected intraperitoneally 5 minutes prior to illumination. Subsequently, the exposed area was illuminated through the intact skull. After 8.5 minutes of illumination, light exposure was stopped. For postoperative care, all animals received analgesics (Novalgin, Sanofi) for at least 3 days after surgery.

For constant delivery of antibodies and growth factors, mice were randomly assigned to 4 groups and received: 1) 10 μg/ml recombinant human VEGF_165_ (R&D Systems) (N = 7); 2) 7 mg/ml Ig G1 mouse monoclonal anti-Nogo-A antibody 11C7 (Novartis) (N = 5); (3) combinational 10 μg/ml VEGF and 7 mg/ml anti-Nogo-A Ab (n = 7); or (4) 7 mg/ml of the control antibody Ig G1 isotype FG12 (n = 6) through implanted mini-osmotic pumps for 7 consecutive days (Alzet Model 107D, Alzet Brain Infusion Kit 3). The concentrations were chosen based on previous studies^23,24^. The cannula of the pump was inserted into the left ventricle (anterior/posterior −0.57 mm, medial/lateral −1.5 mm, dorsal/ventral −2.1 mm to Bregma). The osmotic pump was placed in the midscapula region to permit free head and neck movement. Although the osmotic pump begins operating immediately, we adapted the set-up to allow a 24 h delay of the application. This was achieved by filling the tip of the catheter with a control solution (0.9% saline) according to the manufacturer’s recommendations.

### EdU application

In order to identify proliferating endothelial cells, the animals received three consecutive i.p. injections of 5-ethynyl-2′-deoxyuridine (EdU, Sigma) at a dose of 50 mg/kg on day 6, 7 and 8 after stroke. EdU was detected using the Click-iT EdU Alexa Fluor 647 Imaging kit (Thermo Fisher Scientific) according to the manufacturer’s protocol. Endothelial cells (CD31^+^) incorporating EdU were considered as newly formed following stroke.

### Tissue processing

Mice were deeply anesthetized by intraperitoneal injection of pentobarbital (150 mg/kg body weight, Streuli Pharma AG). Animals were perfused with isotonic Ringer solution (containing 5 ml/l Heparin, B. Braun) followed by paraformaldehyde (PFA, 4%, in 0.2 M phosphate buffer, pH 7). For immunohistological analysis, brains were removed and 4 h post-fixed in 4% PFA, transferred to 30% sucrose for cryoprotection and stored at 4 °C. Coronal sections with a thickness of 40 µm were cut using a sliding microtome (Microm HM430, Leica). Sections were collected and stored as free-floating sections in cryoprotectant solution at −20 °C until further processing. For spectrophotometric analysis of CNS tissue was isolated and stored at −20 °C before further processing.

### Immunohistochemistry

For immunohistochemistry, brain sections were washed with 0.1 M phosphate buffer (PB) and then incubated for 30 minutes at room temperature in a blocking solution containing TNB, TBST (0.1%) and normal goat serum (3%). For detection of vascular endothelial cells, sections were incubated overnight at 4 °C with monoclonal rat anti-CD31 antibody (BD Biosciences, 1:50). Tight junction proteins were detected with the following antibodies: mouse anti-Claudin-5 antibody (1:200, ThermoFischer); rat anti-VE-Cadherin antibody (1:100, ThermoFischer) and rabbit anti-ZO-1 antibody (1:100, ThermoFischer). Pericytes were visualized with goat anti-CD13 (1:200; R&D). The primary antibody incubation was followed by 2 h incubation at room temperature with corresponding fluorescence secondary antibodies (1:500, ThermoFisher). DAPI (1:2000 in 0.1 M PB, Sigma) was used to visualize nuclei. Sections were mounted in 0.1 M PB on Superfrost PlusTM microscope slides and coverslipped using Mowiol.

### Evans Blue quantification to assess systemic leakage

EB dye is an inert tracer commonly used for investigations of vascular permeability in animal models. EB dye binds to serum albumin. Since serum albumin does not cross the BBB under normal physiological conditions, spectrophotometric determination of EB dye accumulation in brain tissue outside blood vessels reflects the extent of vascular leakage.

Tissue samples, which were collected as previously described, were homogenized in lysis buffer (250 μl/mg tissue weight; Tris-HCl, EDTA, NP-40, NaCl, protease inhibitor cocktail) and incubated for 2 hours at 4 °C, shacking. The mixture was centrifuged to remove precipitated fragments (25 min, 15000 g, 4 °C) and the extracted supernatant was collected in a 96-well plate. The EB concentrations were measured at 620 nm using a standard microplate reader (Spark, Tecan) and quantified against a standard curve. The results were expressed as micrograms of EB per milligram of brain tissue.

### Fluorescence microscopy and quantification

Imaging of brain sections 10 days after stroke was performed with an Axio Scan.Z1 slide scanner (Zeiss) and an Olympus FV1000 laser scanning confocal microscope equipped with 10×, 20× and 40× objectives. Images were processed using Fiji (ImageJ) and Adobe Illustrator CS6. First, stroke sizes were compared between animals in 40 μm coronal sections stained with fluorescent NeuroTrace (1:500, ThermoFischer) and DAPI (1:2000, Sigma). Brain sections at sic defined landmarks (2.5, 1.5, 0.5, −0.5, −1.5, −2.5 mm in relation to bregma) were analyzed for depth of the cortical lesion. The dorso-ventral, medio-lateral, and anterior-posterior stroke extent was then used to modulate a precise ellipsoid with the coordinates relative to Bregma.

All other analysis steps were performed in the same region of interest (ROI), a peri-infarct region distal to the stroke core with a width of 300 μm. To assess post-stroke angiogenesis an ImageJ (Fiji) script was established to automatically calculate (1) area fraction of blood vessels, (2) number of blood vessels, (3) number of branches and junctions, (4) distance between blood vessels and (5) vessel distribution^36^.

### Statistical analysis

Statistical analysis was performed using RStudio. Sample sizes were designed with adequate power according to our previous studies and to the literature. All data were tested for normal distribution by using the Shapiro-Wilk test. Multiple Normally distributed data were tested for differences with a two-tailed unpaired one-sample t-test to compare differences between two groups. Non-normally distributed data were tested with a Mann-Whitney U test. Multiple comparisons were initially tested for normal distribution with the Shapiro-Wilk test. The significance of mean differences between normally distributed multiple comparisons was assessed using Tukey’s HSD. Non-normally distributed multiple comparison data were tested by Dunn’s test. Data are expressed as mean ± SD and statistical significance was defined as *p < 0.05, **p < 0.01, and ***p < 0.001.

## Supplementary information

SUPPLEMENTARY INFO.

## Data Availability

All raw data is available upon request.
