# Supplementary material for: Anti-Nogo-A antibodies prevent vascular leakage and act as pro-angiogenic factors following stroke
Source: Sci Rep. 2019 Dec 27;9:20040. doi: 10.1038/s41598-019-56634-1 (PMC6934709; doi:10.1038/s41598-019-56634-1)
Supplement: Supplementary file 1 — SUPPLEMENTARY INFO. [file 41598_2019_56634_MOESM1_ESM.pdf]

Research Article: Supplementary Information

Anti-Nogo-A antibodies prevent vascular leakage and act as pro-angiogenic factors following stroke

Ruslan Rust[1,2], Rebecca Z. Weber[3], Lisa Grönnert[1], Geertje Mulders[2], Michael A. Maurer[1], Anna-Sophie Hofer[1,2], Andrea M. Sartori[1,2], Martin E. Schwab[1,2]

[1] Institute for Regenerative Medicine, University of Zurich, 8952 Schlieren, Zurich, Switzerland

[2] Dept. of Health Sciences and Technology, ETH Zurich, 8092 Zurich, Switzerland

[3] Dept. of Biology, ETH Zurich, 8093 Zurich, Switzerland

Correspondence:

Ruslan Rust
Postdoctoral Researcher
Stem Cell-based Disease Modeling and Regenerative Therapies
Wagistrasse 12
CH-8952 Schlieren / Zurich, Switzerland
email: ruslan.rust@irem.uzh.ch
phone: +41 44 635 7682

Funding:

-

Running headline:

Anti-Nogo-A antibodies prevent vascular leakage

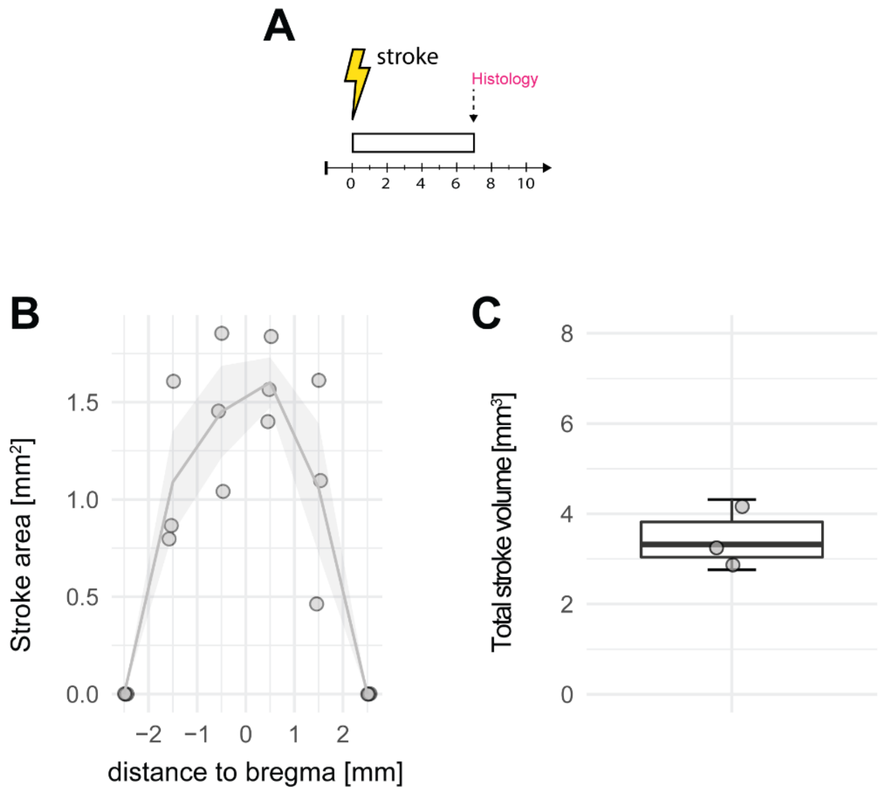

Figure S1: (A) Schematic overview of experimental time line. (B) Measurement of total stroke area and (C) total stroke volume seven days following stroke induction. Data are mean ± SD. Each dot in the plots represents one animal.

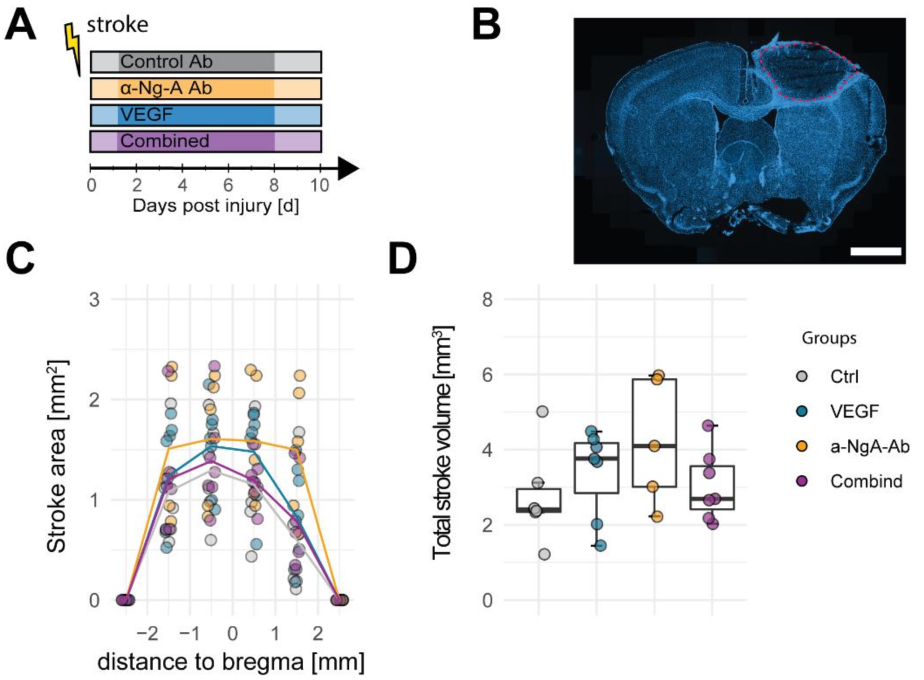

Figure S2: (A) Schematic overview of experimental time line. (B) Representative image showing coronal brain section of stroke mouse visualized with NeuroTrace ten days following stroke. Scale Bar: 1 mm. (C) Measurement of total stroke area and (D) total stroke volume in animals treated with Ctrl Ab, VEGF, a-Ng-Ab and combined treatment 10 days after stroke. Data are mean ± SD. Each dot in the plots represents one animal and significance of mean differences between the groups was assessed using Tukey's HSD.
